# Factors and Mechanism Influencing Client Experience of Residential Integrated Health and Social Care for Older People: A Qualitative Model in Chinese Institutional Settings

**DOI:** 10.3390/ijerph20054638

**Published:** 2023-03-06

**Authors:** Wenya Zhang, Xiaojiao He, Zhihan Liu

**Affiliations:** 1 Center for Social Security Studies, Wuhan University, Wuhan 430072, China; 2 School of Public Administration, Central South University, Changsha 410075, China

**Keywords:** integrated health and social care, older people, client experience, grounded theory, influence mechanism

## Abstract

Background: An emerging service delivery model of integrating health and social care for older people has been actively promoted by the Chinese government since 2016, but the client experience and influencing mechanism still remain unclear. Methods: this study adopts a qualitative methodology to delve deeper into the factors and mechanism shaping the client experience of residential integrated health and social care for older people in the Chinese context, so as to understand the experiences of older residents during the whole process of receiving integrated care services, and on this basis, put forward suggestions for the improvement of a high-quality aged care service system. We coded and analyzed the in-depth interview data of twenty older adults and six staff members from June 2019 to February 2020, recruited from six institutions in Changsha, one of the ninety pilot cities for integrated health and social care in China. Results: the findings showed that the client experience of older adults is mainly affected by factors in three dimensions (scene construction, individual minds, and interaction and communication), which are comprised of six sub-categories (social foundation, institutional functions, perception and emotion, cognition and understanding, intimacy and trust, and participation). Based on the factors and mechanism (consisting of six influencing paths), we constructed a model of the client experience of integrated health and social care for older people in the Chinese population. Conclusions: the factors and mechanism influencing the client experience of integrated health and social care for older people are complex and multifaceted. Attention should be paid to the direct effects of perception and emotion, institutional functions, intimacy and trust in the client experience, and the indirect effects of social foundation and participation on the client experience.

## 1. Introduction

China is currently experiencing a surge in its aging population, which continues to increase rapidly. At the end of 2021, 14.2% of the population were aged 65 years and older, up from 7% in 2000 [1,2]. It is expected that the population of old adults will exceed 400 million in 2033, and reach a peak of 487 million in 2053, accounting for a quarter of the global older people’s population. The old adults’ demand for health, medical treatment, rehabilitation, nursing, and other services is constantly increasing [3]. Given the widespread coexistence of functional decline and multiple chronic health conditions in older people, the traditional pattern of aged care in China, relying on social care delivered by family members, volunteers, or nursing homes, has been unable to meet their needs. There is an urgent need to integrate medical and non-medical care resources to improve service capacity and quality for older adults. Some well-known initiatives/programs applying this concept have already been established in developed countries for years, such as PACE (Program of All-Inclusive Care for the Elderly) [4] and CBSS (Community-Based Supports and Services for Older Adults) [5] in the United States, the aged care reforms in Australia, and ‘Embrace’ [6] in the Netherlands. In 2020, the World Health Organization launched the ICOPE (Integrated Care for Older People) implementation pilot program, which aimed at delivering integrated and person-centered care for older people, in recognition of the need for a transformation of health and social care systems and services.

Although starting at a relatively late stage, China has also begun pilot work in this area. In 2013, the State Council of China promulgated ‘Several Opinions on Accelerating the Development of the Elderly Care Service Industry’ (GF[2013]No. 35), which officially pointed out, for the first time, that a service mode delivering integrated health and social care for older people—referred to as ‘*Yiyang Jiehe*’ in Chinese—was scheduled to be actively promoted [7]. Since 2016, China has set up 90 national-level pilot cities for this service mode. China’s integrated health and social care system for older people is rapidly developing in three primary ways: (a) expanding existing institutions. This consists of two sub-types, the first of which involves adding health care to aged care units. The second sub-type involves adding aged care to health care units. (b) Joint operation. This is based on the framework of cooperation agreements signed by health and social care units. In this way, health care providers regularly deliver health-related services, such as common disease screening and diagnosis, chronic disease management, etc. (c) Holistic home- and community-based care. This delivers family doctor-type services, such as establishing sickbeds in home settings, and primary care staff providing home visits to older people in need. 

In addition to changes in policies and practices, Chinese research has been enhanced by the international concept of ‘integrated care’. Since the first piece of Chinese academic literature with the theme of ‘*Yiyang Jiehe*’ was published in 2005 [8], related research has developed rapidly in China. Some bibliometric analyses employing CiteSpace showed that, since 2004, there has been a steep growth in the amount of research on integrated health and social care for older people in China [9].

However, most studies have focused on expounding the theoretical basis of the integrated service delivery models [10], analyzing and evaluating supply and demand [11], or proposing optimization mechanisms for theoretical operation [12]. Chinese academic research on the integration of health care and social care for older people was intended to extend beyond macro-level theoretical analysis, and begin to search for empirical evidence and practical solutions at the meso and micro levels. Furthermore, there has been a striking lack of analysis and evaluation of the system performance of this emerging service delivery mode through the lens of older people as service recipients, especially based on evidence from China.

The perspective of service recipients should be valued more highly in integrated care for older people, focusing on the following two aspects. First, the older adults’ individual experiences form a core value of person-centeredness in care [13]. The client experience data could play a key role, not only in evaluating the overall performance of the integrated care system, but also in improving service quality and accountability, and redesigning care delivery [14,15,16]. Second, the nature of public services, and the increasing consensus on co-production in integrated health and social care programs, calls for involving clients in the design and monitoring of services [17,18].

Research on customer-value orientation and the quality-of-care orientation have different definitions of the client experience, but they all focus on service recipients’ subjective feelings and judgments during and after the journey through the care service system [19]. Although health care does have many particularities, it is, ‘after all, a service’ [20,21]. Several recent studies have integrated these two approaches, to form a conceptual framework of the older adults’ experience, which contends that an individual becomes a patient at the onset of disease, and a service user or customer in the first contact/interaction with health care services [21]. Quantitative research continues to predominate in the field of the client experience of integrated aged care, especially in China. These studies have mainly adopted cross-sectional study design and satisfaction instruments to evaluate older individuals’ experience of health care services, lacking dynamic consideration of service processes and recipients’ psychological changes across the continuum of care. Therefore, the current study employed a qualitative methodology to gain an in-depth understanding of the experiences of institutional older people during the whole process of receiving integrated care services. The analysis explores the influencing factors and mechanism of the client experience of older adults in the context of China, in order to put forward suggestions for the improvement of a high-quality aged care service system.

## 2. Materials and Methods

This research was designed to explore the influencing factors and mechanism of the service experience of integrated health and social care for older people, based on the qualitative method of grounded theory. This method searches for the core concepts reflecting social phenomena in the systematic collection of data, and then constructs theories from the bottom up through exploring the connections among these concepts [22]. Compared with other qualitative methods emphasizing description, grounded theory pays more attention to the construction of theories [23].

### 2.1. Sample Selection

The study sample was taken from institutions delivering integrated health and social care to older people in Changsha City, Hunan Province, China. Hunan is one of the most aging provinces in China. In 2000, Hunan formally became an aging society, and since then, the aging rate has accelerated. In 2020, 14.8% of the total population in Hunan Province were older people aged 65 and over, an increase of 5.04% and 7.37%, respectively, compared with 2010 and 2000. As the provincial capital city of Hunan, Changsha was selected as one of the initial national pilot cities of integrated care for older people in 2016, and has made remarkable achievements in the ‘expanding existing institutions’ mode of health and social care integration. In the list submitted to the central government in 2022, for the election of national-level demonstration institutions of integrated health and social care for older people, the Changsha region accounted for five out of nineteen institutions in Hunan.

In accordance with the principle of theoretical sampling advocated by the grounded theory method [24,25], from June 2019 to February 2020, we conducted a two-step survey in Changsha. First, based on a review of the literature and visits to health and civil affairs departments in Hunan Province and Changsha City, we determined a sampling frame and selected six institutions in which to carry out the survey. As there are three main types of integrated care institutions for older people in China, nursing home, assisted living community, and residential care facility [26], the institutions were selected based on their types at first. In the meantime, the chosen institutions indicated a willingness to participate, along with diversity, and a reputation for delivering integrated health and social care. During this process, we combined stratified sampling and snowball sampling, considering regional distribution, institutional categories, and data availability. For instance, when a nursing home was chosen and the investigation was completed, it was required to recommend another integrated aged care facility of the same type. The recommended institution was supposed to be geographically separated from the previously completed sample. The research team continually examined the theoretical saturation of data in the subsequent interviews. After two rounds of sampling, the theoretical saturation was found to be reached when six institutions were investigated. Second, we employed random sampling and snowball sampling to recruit suitable older individuals among these institutions’ residents. The inclusion criteria were as follows: (1) ≥60 years old; (2) no significant hearing or vision impairment; (3) basic cognitive ability and communication ability to participate in in-depth interviews; (4) having used at least one type of integrated health and social service in the sampled institutions for more than one month. A total of twenty older adults from the six sampled institutions, across two institutional settings, formed the final sample. The number of samples was determined according to the principle of theoretical saturation, where sampling continues until the new sample no longer provides new information.

### 2.2. Data Collection

In this study, face-to-face in-depth interviews were used to collect data. The interviewers were five senior postgraduate students under the supervision of a professor in Social Medicine and Health Care Management. The research team had received training in grounded theory and communication skills. According to the requirements of grounded theory, the interviewers did not begin with a fixed, strict, and unified outline. The five interviewers independently conducted the interviews without any interferences or presets. During the interviews, they did not interrupt the speech of older residents, and avoided inducing behaviors. Therefore, the data collection process consisted of two phases. The first one was a small-scale preliminary interview, mainly inquiring about the most urgent needs of older people, their feelings about the health and social care services delivered by the institutions, and their satisfaction with the national support policies for older people. The feedback from this phase was used to determine whether the scope of the existing interview questions needed to be widened, or if a new group of interviewees in other institutional settings needed to be involved. Thus, in the second phase, a detailed investigation of the older individuals’ family situation, economic conditions, social status, mental health, and interpersonal communication was added to the interview (the final version of the interview schedule is available in the Supplementary Materials).

To ensure interviewees’ right to know the details of the data collection, after obtaining informed consent from the participants, the interviewers clearly informed each interviewee of the purpose and significance of the interview, and of the use and protection methods of the data. In addition, the study also adhered to the voluntary principle of participation. Each staff member was interviewed for approximately 30 min and had received permission from their supervisors. Their supervisors did not intervene or interrupt while the staff member was being interviewed. The names of the interviewees and institutions were saved in the form of codes. To avoid interviewees making any unnecessary presuppositions, the interviewers only played the role of explaining the theme and deepening exploration of the topic during the process; interviewees were allowed to express their opinions as much as possible until they ran out of content to discuss. Each interview lasted for approximately 30–60 min, depending on the individual situation. Each interview was recorded to ensure that no information was omitted. The recordings were transcribed into text form within two days, with one researcher transcribing the data and another checking the transcription. When inconsistent results occurred during the transcription process, communicative validation was performed according to the following three principles: (1) coding the inconsistent results separately, and then discussing them together; (2) making judgments based on opinions of a third person when there is a conflict of views; (3) referring to the literature. All the information was synthesized into a verbatim manuscript of more than 70,000 words. One researcher interpreted the meaning of the data through constant comparisons, gradually reducing, transforming, and abstracting the original data into higher-level concepts and categories, and proposing a conceptual framework through in-depth analysis of the nature, characteristics, and relations of the categories. In the entire process, the data were compared, summarized, and deduced in a circular manner to form a theory that conforms to the original materials. The research team agreed upon the interpretation of the results.

### 2.3. Reliability and Validity

Credibility and dependability have been used to describe various aspects of trustworthiness in qualitative content research [27]. We believe that, in addition to the elaboration of clients’ perspectives, practitioners’ perspectives should also be explored. Therefore, in order to ensure the credibility of the collected data, an interview was conducted with a staff member randomly selected from each sampled institution, who has close contact with older individuals, and directly participates in the service delivery.

The encoding reliability test method, advocated by Huberman and Miles, was adopted in this study, and the scientific reliability of the encoding results was confirmed through analysis of the coding consistency percentages of two independent coders [28]. The specific process involved randomly selecting two copies of the verbatim transcripts, another coder re-coding them according to the coding scheme, comparing the two coding results, and calculating the number of mutual agreements and disagreements between the two coders. Through this calculation, the reliability was found to be 90.53%, and the internal consistency was 94.62%, indicating that all the coding had good reliability.

Furthermore, the research team invited two professors of Social Medicine and Health Management and two care managers at the integrated aged care institutions to evaluate the degree of consistency between the results and the actual situation. Their positive feedback verified the high validity of the research results.

### 2.4. Data Analysis and Processing

We used NVivo 11.0 to perform auxiliary coding and analysis. Given the interviewees’ linguistic expression conventions, and the format of the data structure, sometimes multiple lines of data were needed to express a complete concept or category [29]. Therefore, this study did not strictly adhere to the principle of ‘line-by-line coding’ required by grounded theory [30]. Rather, ‘word-by-word coding’, ‘sentence-by-sentence coding’, ‘line-by-line coding’, and ‘paragraph-by-paragraph coding’ were used comprehensively to code the interview data, according to the three-level coding method of grounded theory proposed by Strauss and Corbin [31].

To start the data analysis, first, we applied concept naming to all the original material. Then, we merged similar concepts, establishing the ascription between phenomenon and categories. Some ambiguous or overlapping concepts had to be repeatedly compared and analyzed to complete the categorization. Next, we discovered and established logical connections between the categories by categorizing and analyzing the scattered data and integrating them again. Selective coding is the final step, which organizes the data and discovers the core category by describing the ‘storyline’ of the phenomenon [32].

## 3. Results

### 3.1. Information Regarding Socio-Demographic Data of the Participants

A total of twenty older adults from the six sampled institutions, across three institutional settings, formed the final sample (see Table 1 and Table 2 for a description of the sample characteristics). As shown in Table 1, the average age of older adults living in institutions is 79.7 years old (ranging from 68 to 94 years old), among which, the proportion of females (70%) is much higher than that of males (30%), and the educational level is concentrated in those with a high school education or below (90%). In addition, through interviews, we learned that the aged care service institutions in Changsha set tiered charging standards, generally based on the physical conditions of older adults. The basic service fees of public aged care service institutions are managed by the government with a guided price, ranging from CNY 2000 to 5000, while the fees of other institutions are slightly higher, mostly more than CNY 3000. The monthly income of most older people is less than CNY 3000 (65%), which makes it difficult for them to fully cover the cost of institutionalization, and the difference is mostly paid by family members.

As shown in Table 2, most of the staff members are female (83%) and older. Most of them come from rural areas, with a low educational level (83%), and they also lack vocational nursing skills training. The form of employment is mostly in the form of temporary and contract workers, so the wages are relatively low, mostly concentrated around CNY 3000.

### 3.2. Information Regarding Analysis Based on the Qualitative Method of Grounded Theory

Through the process of developing the concept and refining the categories by open coding, we obtained a final total of 28 categories, based on which spindle coding was performed. By integrating the scattered data again, and identifying the logical connection between the categories, a total of three main categories and seven sub-categories were summarized (Table 3). The specific coding rules for the interview records are shown in Table 4.

In our study, ‘influencing factors of the client experience of integrated health and social care for older people’ was defined as the core category, which is composed of three main categories: scene construction, individual cognition, and interactive communication. Thus, the related ‘storyline’ can be summarized as follows: ‘the client experience of integrated health and social care for older people is mainly affected by scene construction, individual minds, and interaction and communication’. According to the storyline, the typical relationship of the factors affecting the client experience of integrated aged care for old people in China was initially constructed (Table 5).

**Figure 1:**
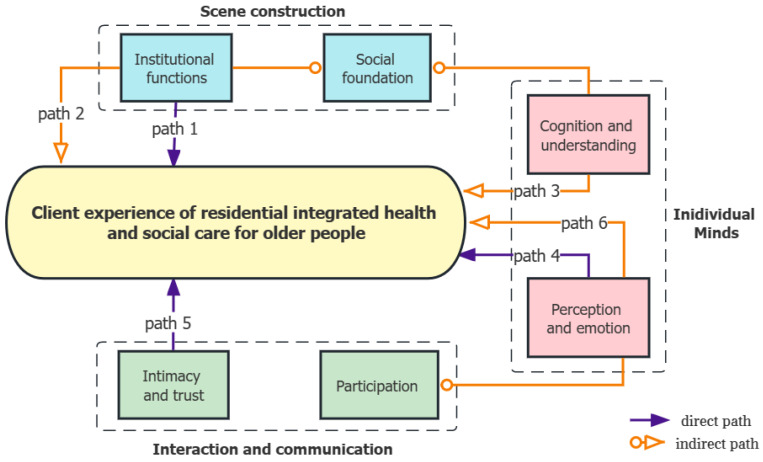
A model of the factors and mechanism of the client experience of integrated care for older people.

Based on the detected relationships among categories, we constructed a model of the influencing factors and mechanism of the client experience of integrated aged care, as shown in Figure 1. The model includes three dimensions (main categories)—scene construction, individual minds, and interaction and communication—and six factors (sub-categories), among which there are six influencing paths.

#### 3.2.1. Influencing Path 1: Institutional Functions → Client Experience

The nature and scale of institutions, to a certain extent, determine the object, scope, and level of the services provided. The government-led public aged care institutions are generally favored by older Chinese, due to their low fees and good reputation, but the amount of beds is rather limited. Some older people who cannot find a provider are prioritized for admission, as stipulated by the government, meaning that such institutions are often oversubscribed in China. Many older people have to turn to private institutions, some of which struggle to carry out diversified service projects, due to limited capital and space. However, such institutions tend to look for other ways to compensate for the deficiencies caused by insufficient service supply, such as enhancing brand characteristics in the facility environment, or entertainment activities.

It is worth noting that the living environment has become an important factor influencing the client experience of older individuals, from the traditional client perspective [33,34]. Institutions which allow older people to decorate their rooms according to their own wishes, and create a warm environment similar to home, have been found to result in a more positive client experience than institutions that smell of disinfectant, like a hospital. For example, an involved older adult said:

*‘They didn’t let me put pictures on the wall or table, saying it was unsafe... My husband has passed away. This is a group photo of us, and I would like to watch it many times every day.’ (KNa19)*

#### 3.2.2. Influencing Path 2: Social Foundation → Institutional Functions → Client Experience

The social foundation includes the all-round support of the economy, politics, and culture. It directly promotes the development of integrated health and social care for older people, but it has a more indirect impact on older adults’ experience, mainly through improving the service delivery of integrated health and social care [35]. Systemic flaws may hinder the promotion of policy. The Ministry of Civil Affairs, as the administrative department in charge of nursing homes and other aged care institutions, faces difficulties in effectively coordinating with other departments in charge of the health care system, such as the Health Ministry and the National Medical Insurance Bureau, which have caused some policies to be implemented inadequately. Moreover, the aged care service is only a part of the civil affairs. In the case of limited resources, there are differences in the degree of policy support enjoyed by integrated aged care institutions of a different nature and size.

*‘…not implemented… it is just not implemented by the low-level governments. There is an example: For people over 80 years old, their pensions are tens of yuan (each month) … The money in the last quarter has to be paid in the next quarter. What is the trick? Look, it’s like a game. The money in December has not arrived yet. It will not arrive until February. What do you think the trick this is? What can I do? The government is just like this… Do you think the leader is aware of this? Does he know? Nobody tells him.’ (YFa10)*

#### 3.2.3. Influencing Path 3: Social Foundation → Cognition and Understanding → Client Experience

The social context is a part of the cognition system and cannot exist independently of cognitive activities. Compared with the macro context of the institutional system and financial investment, older people pay more attention to media reports and public evaluation, things they can access on integrated health and social care. Diversified modes of news communication are widely favored by older people, which to a large extent meet their requirements for leisure, entertainment, and daily communication convenience. The interpretation of integrated care policies by the media, and the reports of related events, could shape and affect their cognition, particularly if some biased news reports and comments mislead them. The older people bring their fragmented cognition into the service process, and compare the reality with their subjective understanding. They become constructors of information, and influence the group’s experience or satisfaction through their relationship network. For example, one older adult stated:

*‘You can see on the mobile phone that the babysitter steals things and abuses the older people all day long. Many children don’t know about it, and they have to install monitors to find it. There is no filial son in front of the bed after a long illness. If your own children are unwilling to serve you, how can they be sincere to you?’ (HRa16)*

#### 3.2.4. Influencing Path 4: Perception and Emotion → Client Experience

Older adults’ experience of integrated health and social care is related to their inner perception and emotion—the two factors have been proven to be more inseparable than is commonly assumed [36]—before and after being enrolled in the service system, as services are not as visible and easy to standardize as goods. At present, China’s integrated health and social care for older people is still in the exploration phase, and there is uneven development among the various institutions. Institutions that pay more attention to relatively observable attributes of service (responsiveness and attitude of personnel, continuity of service, etc.), which positively or negatively affect older adults’ perception and judgment of service quality [33,37] and emotion, receive significantly better client experience evaluations. Some participants recounted that:

*‘We can accept the bad living conditions, but the staff must be good to us, which is the most important thing. Service belongs to non-physical configuration while living conditions belong to physical configuration (of the institution). If there is only physical configuration and no non-physical configuration (here), the residence will not be interesting.’ (LLa01)*

*‘Some old people want to go home, and their children take them back once a month. Well, I am alone. I cannot move or go home now, and everything is taken care of by them. Their services basically cover all aspects of my life, but sometimes I feel really lonely (choking…).’ (Hra16)*

#### 3.2.5. Influencing Path 5: Intimacy and Trust → Client Experience

The client experience of integrated health and social care for older people takes services or service behaviors as the carriers, and is jointly manifested through the interaction between service providers and recipients. It is worth noting that although interaction between staff and clients has been widely recognized as one of the key factors contributing to the client experience [38], we found slight differences in the way staff interact with older people at different stages. Generally, older people will have a period of adaptation after they have just settled in at an integrated aged care institution. During this period, staff need to focus on psychological comfort and debugging for older people, to help them adapt to their new lives as soon as possible. At this time, the interaction is more one-way, consisting of staff output. Once the older people have gone through the period of adaptation, they might receive less attention from staff, due to the limited numbers and heavy workload of staff members. Older people will actively seek to interact with the staff and older people around them, and make the interaction a two-way process of service information feedback and communication.

When this interaction results in an intimate interpersonal relationship, the services delivered by the staff and institution will be more likely to gain credibility. In addition to forms of interaction, such as co-building WeChat groups with medical personnel (as in the example in Table 3), the personal characteristics of managers, their leadership style, management philosophy, and understanding of policies, will also exert an influence on the experience of older people. The interviews showed that most managers of aged care institutions (especially the private type) know better than their peers in other industries to remain humble and charismatic in interactions with seniors. This not only affects older people’s sense of intimacy and trust, but also affects the attitude and behaviors of staff [39]. For example, an interviewee noted the following:

*‘Monitor Peng is a real warm-hearted man. He is the labor chief and a veteran Party member. Those surrounding communities rely on him the most. The communities are engaged in garbage sorting these days and he is on duty at 7 o’clock every morning. Young people can’t do it.’ (FLa07)*

#### 3.2.6. Influencing Path 6: Participation → Perception and Emotion → Client Experience

Most studies have shown that customer participation in service production is conducive to creating happy experiences and meeting their personalized needs [40]. More often, the interactions between older people and service providers are aimed at emotional communication, rather than satisfying service demand. Even if the problem that may cause a negative experience is not eventually solved, as long as the older people feel engaged in the quality improvement process based on their feedback, their experience, satisfaction, and even loyalty could be enhanced [41]. Participation may provide older people with self-confidence, a sense of belonging and being respected, pleasure, psychological comfort, etc., which may also lead to a positive client experience. One interviewee mentioned:

*‘Last time, I said that the food was too bland and tasteless. She told me that she did not dare to make it too salty. “Relatively mild-flavored food is good for your health, and you should take care of the taste of most seniors”, she told me that, and I understood. She said that she could cook me some slightly more flavorful meals alone, but I would definitely not accept it. It will undoubtedly increase the workload of others. After all, I am not living in my own home, and there are so many older people living here.’ (YJa13)*

## 4. Discussion

Aims at understanding the experience of older people during the whole process of receiving residential integrated care services and making policy proposals, this study adopts a qualitative methodology to delve deeper into the factors and mechanism shaping the client experience of residential integrated health and social care for older people in the Chinese context. Overall, we found that the vast majority of older people were satisfied with their experience of integrated health and social care. Even some small-scale institutions with limited health care facilities also received high praise from older people, because such institutions tend to make improvements to the non-physical aspect of care delivery to compensate for shortcomings in the physical aspect.

This study identified six factors in three dimensions (scene construction, individual minds, interaction and communication) affecting the client experience of integrated health and social care for older people, and revealed how they function in the Chinese context. The results are consistent with the research of Jiun-Sheng Chris Lin et al. (2016), and generally cover the seven topics of service experience they summarized: customer/employee emotion, service employee management, service environment, customer participation, self-service technologies, service failure/recovery, and customer loyalty management [42]. In addition, one of the few similar qualitative studies published in Chinese academic journals argues that there are four themes influencing older adults’ experience of integrated care: policy, manpower, nursing care delivery, and medical insurance qualification [43]. In the field of service users’ satisfaction and experience of aged care, more quantitative research has been conducted, whose conclusions focus on the factors causing heterogeneity in older individuals and service providers, paying less attention to the situational factors at the level of policy, economy, culture, etc. [44]. This study differs from existing studies in that its findings not only cover the heterogeneity of both sides of the service, but also take into account situational factors with Chinese characteristics.

### 4.1. Dimension of Scene Construction

This research shows that the self-reported experience of institutional integrated health and social care for older people is grounded in the scene construction of care delivery. There are numerous critical factors in institutional characteristics, such as size, location, level, service content, etc., which all influence residents’ experience [45]. Studies have validated that the level of care facilities and the service quality of caregivers are important factors affecting client experience [46]. In addition, previous studies have argued that mitigating physical and spatial constraints, and offering immersion into the desired environment and situation, could contribute substantially to the health and experience of older residents in institutions [47]. Similarly, the current study showed that the internal layout and decor of institutions matters to older people. Providing older people with a certain degree of permission to decorate their rooms at will, bringing comfort and familiarity to their living environment, could result in a positive client experience for older residents in integrated care institutions.

We also discovered that China’s social foundation—including factors of political and economic support, atmosphere of public opinion, and contradictions existing in the structure of the implementation system—indirectly impact the client experience of integrated health and social care for older people through institutional functions, such as the scale of institutions, layout of the living environment, service content, and brand image. In the Chinese context, the functions of integrated care institutions are shaped by medical and long-term care insurance policies, land-use planning, etc. Studies in China have likewise confirmed the causal relationship between policies and service quality of institutional care [48]. Preferential policies according to local conditions, reform of the medical and health services system, and encouragement of non-governmental sector engagement, contribute to enhancing the service content, which in turn affects the institutional functions [49]. Moreover, the public’s belief in institutional aged care also has an indirect influence on the client experience of integrated health and social care for older people. Public institutions of integrated care are often the first choice for older adults and their families who are considering institutional care in China, because the public generally believe that they deliver higher-quality and safer health care. However, although these institutions have relatively sufficient and stable sources of funds, they must accept a certain proportion of older adults who are widowed and childless, as stipulated by the government. During the economic downturn and pandemic, this has indeed guaranteed a source of stable income, but the governmental funds are far from enough to cover all the costs for those older adults. As a result, institutions intend to balance this by accepting more self-paid older people (usually a 1:1 ratio is preferred), which may lead to a decline in the resources available to each older individual, and result in negative experiences.

### 4.2. Dimension of Interaction and Communication

Consistent with the existing studies (mostly focused on the private sector rather than the public sector), we also found that the service encounters between older adults and staff are crucial to the evaluation of service experience across the care continuum [50]. Older adults form a judgment of whether the service providers are trustworthy and close enough to them, based on the attitudes and behaviors of the staff at each moment of interaction, and they construct part of the client experience on this basis. They acquire information and support from staff through trusting and continuous relationships, as Facchinetti et al. concluded [51].

In addition, older adults will also take the rate and nature of interaction with staff as important indicators in the evaluation of client experience. Under the condition of relatively limited care capacity, most older people are eager to receive as much attention as possible from service providers. This attention may be expected to provide timely solutions to problems reported by service users, as evidence of their participation in the process of improving the quality of care. On the other hand, inadequate participation demand and ability of older adults may hinder the staff–resident interaction in the institutional setting. From the perspective of staff, they prefer to interact more with residents with a higher degree of mental functioning, rather than with residents with a lesser degree of mental functioning [52].

### 4.3. Dimension of Individual Minds

Cognition is constructed on a social foundation. This study discovered that the client experience of older people is influenced by cognition and understanding based on a social foundation. For example, the social evaluation of aged care institutions influences the choice of institutions for older people. Some previous studies have suggested that private nursing homes tend to be more popular with older Chinese people because of their better social reputation, evaluation, and publicity than their public counterparts [53]. However, our research shows that public care institutions are the first choice for older Chinese people and their families in need of integrated care. Our interpretation of this difference is that when it comes to integrated care for older people, the key to the experience and outcome of service delivery lies in the capability of medical sectors, and their connections with non-medical aged care sectors. In China, public-funded medical sectors are generally considered to be technologically capable in leading to better integrated care delivery processes and outcomes. Nevertheless, our findings are consistent with the existing literature, in that the social foundation can indirectly impact the self-reported experience of aged care by influencing the cognition and understanding of service recipients.

This study also confirms that the perception and emotion of older people have a direct effect on client experience. Older people’s perception of services is influenced by their physical health, education level, socioeconomic status (SES), and emotions [54,55]. An interesting phenomenon we observed is that due to emotional factors, older people usually hope that staff members, specifically care assistants, care more about them than they do about the other seniors. Once they notice that the staff take special care in catering to the needs of certain individuals, they might feel that their rights and interests are being neglected. The resulting sense of unfairness (perceived unfairness) would not only lead to adverse effects on their health [56,57], but also cause complaints about the staff. This irrational factor could be moderated by objective predictors, such as physical health, education level, and SES, which are often positively correlated with the service experience of older people.

On this basis, our study verifies that participation of older people can influence the client experience indirectly by affecting their perceptions and emotions. The participatory model that emphasizes collaboration and partnership can lead to changes in the balance of power between staff and clients [58]. This balance makes older people feel valued, and enhances the client satisfaction. This observation might help explain the consistent findings that higher client engagement tends to result in a better client experience [59] and improved service quality [60].

## 5. Limitations and Recommendations

Despite its strengths in initially addressing the factors and mechanism of institutional integrated care experience among older people in China, this study has limitations in the following two aspects. First, the diversified and personalized service needs of older people with different health conditions are supposed to affect the client experience of integrated health and social care, but this study only involved older people with clear cognition and expression, failing to examine the differences in the client experience between physically or mentally disabled older people and other older people. Second, this study employed a constructivist approach, and the proposed theoretical model requires further empirical evidence. The next step is to test the fitness of the model among older adults in China and beyond, through the development of quantitative tools and structural equation modeling.

## 6. Conclusions

Differing from the quantitative research prevalent in the extant literature on the client experience [61], this study used grounded theory to conduct an in-depth exploration of the factors of client experience of residential integrated health and social care for older people in China, so as to understand the experiences of older residents during the whole process of receiving integrated care services. The research constructed an innovative theoretical framework of client experience of integrated health and social care for older people, including dimensions of scene construction, individual minds, and interaction and communication. Specifically, perception and emotion, institutional functions, and intimacy and trust have direct effects on the client experience of integrated health and social care for older people, while social foundation and participation have indirect effects. This work broadens the scope and depth of research on client experience and satisfaction to a certain extent, and provides new insights into the mediating effects in client experience of integrated aged care for future quantitative studies. With the above factors, this study proposes an optimization plan for the improvement of a high-quality aged care service system, to provide a comprehensive reference for government departments and integrated aged care institutions.

First, as the related resources and policies of integrated health and social care for older people are still fragmented and scattered among departments of civil affairs, medical insurance, and health care, etc., China’s government needs to put more effort into reducing the cross-departmental cooperation resistance caused by uneven interest distribution, and making the service delivery smoother and more seamless. Second, the government departments should scientifically arrange the location, space, and facilities of integrated aged care institutions, matching the actual needs of older adults, by consulting and collaborating with the institutions. Institutions and practitioners are supposed to be more concerned about the self-reported experience of older residents, and accurately identify their service needs to strengthen the person-centeredness of the delivered services. Finally, as clients and co-producers, it is necessary for older adults to participate in the policy formulation and analysis, instrument development and validation, and other aspects in improving the quality of institutional integrated care. This, however, requires more authoritative institutional arrangements to guarantee.

## Figures and Tables

**Table 1:** Sample characteristics of the older individuals (n = 20).

Gender	
Female	14 (70%)
Male	6 (30%)
**Age**	
60–69	2 (10%)
70–79	7 (35%)
80–89	9 (45%)
90–99	2 (10%)
**Education**	
illiteracy	3 (15%)
primary school	5 (25%)
junior high school	5 (25%)
senior high school/secondary specialized school	5 (25%)
undergraduate	2 (10%)
**I** **ncome**	
below CNY 2000 (USD 288)	5 (25%)
CNY 2000–3000 (USD 288–432)	8 (40%)
CNY 3000–4000 (USD 432–576)	5 (25%)
over CNY 4000 (USD 576)	2 (10%)
**Institution type**	
**Nursing home**	**9 (45%)**
YF (government-funded)	6 (30%)
YJ (private)	3 (15%)
**Assisted living community**	**6 (30%)**
HR (private)	3 (15%)
LL (private)	3 (15%)
**Residential care facility**	**5 (25%)**
FL (private)	3 (15%)
KN (private)	2 (10%)

**Table 2:** Sample characteristics of the staff members (n = 6).

Gender	
Female	5 (83%)
Male	1 (17%)
**Age**	
30–39	1 (17%)
40–49	1 (17%)
50–59	4 (66%)
**Education**	
illiteracy	0 (0)
primary school	3 (50%)
junior high school	1 (17%)
senior high school/secondary specialized school	1 (17%)
undergraduate	1 (17%)
**I** **ncome**	
below CNY 2000 (USD 288)	0 (0)
CNY 2000–3000 (USD 288–432)	1 (17%)
CNY 3000–4000 (USD 432–576)	3 (50%)
over CNY 4000 (USD 576)	2 (33%)

**Table 3:** Spindle code table.

Main Category	Sub-Category	Open Category			
Scene construction	social foundation	policy support	economic foundation	atmosphere of public opinion	system dilemma
	institutional functions	institution size	living environment	service content	brand image
Individual minds	perception and emotion	expectation and stereotype	service demand	responsiveness	empathy and consolation
	cognition and understanding	mindset	cognition of policy	information notification	individual characteristics
Interaction and communication	intimacy and trust	role-based trust	charisma of leaders	network	discourse expression
	participation	information sharing	feedback	relative deprivation	weighing of interests

**Table 4:** Number of nodes in specific categories.

Analysis Dimension	Influencing Factor	Example of Reference Points
Scene construction	Social foundation Institutional functions	*We are currently in a state of loss, and there is no profit, but the headquarters will provide some subsidies. The Ministry of Civil Affairs provide subsidies based on the number of beds—tens of yuan per month (for each bed)—which are limited. (LLb01)* *We cooperated with the community health center in this activity. It was supposed to be a promotion of ‘family doctors practice in the home setting’. However, the community health center is a little far away from the community, and the doctors do not have so much time to go door-to-door for physical examinations, so it is very difficult to provide on-call services. We changed it to a different form. The doctors offered free consultations in the community at a fixed time each week. (FLb03)*
Individual minds	Perception and emotion Cognition and understanding	*The support worker who just came in has the worst attitude (pointing at the door). (Whispering…) She has only been here for a few days with a particularly bad attitude, and she steals things. After I took traditional Chinese medicine during the day, I went to the washroom more often, so she became impatient… The night shift support worker has a better attitude and has worked (here) for several years. She boils traditional Chinese medicine for me. It is good to eat traditional Chinese medicine, which can get to the root cause of disease. The body will detoxify if you urinate more. (Yfa11)* *I am 91 years old (prolonged tone). What kind of service do I still need? I only blame myself for living too long. Why live so long? Sigh… (Yja13)*
Interaction and communication	Intimacy and trust Participation	*Look, this is the WeChat group called ‘YF Old Baby’ (51 persons), and the medical staff (of the client) are also in this group. My eyes are not good now. I used to watch the WeChat group a lot, but now I dare not be so presumptuous (laughter…). I can only look at it for a while and take a break, and sometimes, I do not look at it for a few days. My mobile phone is an old one, so I use the tablet for WeChat. (Yfa04)* *The overhaul began in May last year. We moved again and again, from the 19th floor to the 17th floor, the 17th floor to the current 20th floor, and the 20th floor back to the 17th floor. She later asked for everyone’s opinion, and said, “you old people don’t want to move, just live here and don’t move”. I’ll just stay here and not moving. It’s too tiring to move around. (Interviewer: Do the staff usually listen to your opinions?) That’s regular, probably… in every month or two, they send us forms and let us fill in our opinions. (Interviewer: Are you satisfied with their staff?) Relatively satisfied. (Yfa04)*

Notes: The number of nodes refers to the number of occurrences in the reference node during the encoding process. Coding rules of interview records: for the sake of respecting the wishes of interviewees, the names of all interviewees are hidden and replaced by a combination of English letters and numbers. The capital letters indicate interview location, and the lowercase letters denote respondent category, where ‘a’ represents older individual, ‘b’ represents staff member, and the number represents the serial number of respondents.

**Table 5:** Typical relationships among influencing factors.

Relationship Connotation	Relationship Structure
social foundation → institutional functions → client experience	indirect influence
institutional functions → client experience	direct influence
social foundation → cognition and understanding → client experience	indirect influence
perception and emotion → client experience	direct influence
intimacy and trust → client experience	direct influence
participation → perception and emotion → client experience	indirect influence

## Data Availability

The datasets generated and analyzed during the current study are not publicly available, to protect the participants’ confidentiality. However, they are available from the corresponding author on reasonable request.

## Supplementary Materials

The following supporting information can be downloaded at: https://www.mdpi.com/article/10.3390/ijerph20054638/s1, File S1: Interview Outlines.
