# Supplementary material for: Factors and Mechanism Influencing Client Experience of Residential Integrated Health and Social Care for Older People: A Qualitative Model in Chinese Institutional Settings
Source: Int J Environ Res Public Health. 2023 Mar 6;20(5):4638. doi: 10.3390/ijerph20054638 (PMC10002276; doi:10.3390/ijerph20054638)
Supplement: Supplementary file 1 [file ijerph-20-04638-s001.zip › ijerph-2201687-supplementary.pdf]

Interview Outlines

With Older Residents:

1. How do you feel about the experience of integrated service delivery of multiple care (e.g., social care, medical care, rehabilitation care) during the residence?
2. Which service delivered do you value more?
3. What is your most urgent service demand? Did it have been met?
4. Which factor do you think has the greatest impact on your experience?
5. What do you think of the current aged care model of integrating health care with social care?
6. What do you think of the national support and development policies for older people?

With Staff Members:

1. Please describe the current situation of the institution's development on the integrated care for older people and the older residents' acceptance of such services.
2. How do older residents always express their service demands and how were them satisfied?
3. Which factor do you think has the greatest impact on older resident experience of residential integrated care?
4. Has the experience of older residents been evaluated formally?
5. What do you think of the current aged care model of integrating health care with social care?
6. What do you think of the national support and development policies for older people?
